# Epigenomic Profiling Advises Therapeutic Potential of Leukotriene Receptor Inhibitors for a Subset of Triple-Negative Breast Tumors

**DOI:** 10.3390/ijms242417343

**Published:** 2023-12-11

**Authors:** Alexey I. Kalinkin, Vladimir O. Sigin, Ekaterina B. Kuznetsova, Ekaterina O. Ignatova, Ilya I. Vinogradov, Maxim I. Vinogradov, Igor Y. Vinogradov, Dmitry V. Zaletaev, Marina V. Nemtsova, Sergey I. Kutsev, Alexander S. Tanas, Vladimir V. Strelnikov

**Affiliations:** 1 Research Centre for Medical Genetics, 115522 Moscow, Russia; sigin.vladimir@gmail.com (V.O.S.); kuznetsova.k@bk.ru (E.B.K.); md.ignatova@gmail.com (E.O.I.); zalnem@mail.ru (D.V.Z.); nemtsova_m_v@mail.ru (M.V.N.); kutsev@mail.ru (S.I.K.); tanas80@gmail.com (A.S.T.); vstrel@list.ru (V.V.S.); 2 Laboratory of Medical Genetics, I.M. Sechenov First Moscow State Medical University (Sechenov University), 119992 Moscow, Russia; 3 Nikolay Nikolaevich Blokhin National Medical Research Center of Oncology, 115478 Moscow, Russia; 4 Regional Clinical Oncology Dispensary, 390011 Ryazan, Russia; ilya_vinogradovonc@yandex.ru; 5 Department of Histology, Pathological Anatomy and Medical Genetics, Ryazan State Medical University, 390026 Ryazan, Russia; maxim_vinogradovonc@yandex.ru (M.I.V.); vinogradovonc@yandex.ru (I.Y.V.)

**Keywords:** breast cancer, leukotriene receptors, DNA methylation, XmaI-RRBS

## Abstract

Triple-negative breast cancer (TNBC) is the most aggressive molecular subtype, with a poor survival rate compared to others subtypes. For a long time, chemotherapy was the only systemic treatment for TNBC, and the identification of actionable molecular targets might ultimately improve the prognosis for TNBC patients. We performed a genome-wide analysis of DNA methylation at CpG islands on a collection of one hundred ten breast carcinoma samples and six normal breast tissue samples using reduced representation bisulfite sequencing with the XmaI restriction enzyme (XmaI-RRBS) and identified a subset of TNBC samples with significant hypomethylation at the *LTB4R/LTB4R2* genes’ CpG islands, including CpG dinucleotides covered with cg12853742 and cg21886367 HumanMethylation 450K microarray probes. Abnormal DNA hypomethylation of this region in TNBC compared to normal samples was confirmed by bisulfite Sanger sequencing. Gene expression generally anticorrelates with promoter methylation, and thus, the promoter hypomethylation detected and confirmed in our study might be revealed as an indirect marker of high *LTB4R/LTB4R2* expression using a simple methylation-sensitive PCR test. Analysis of RNA-seq expression and DNA methylation data from the TCGA dataset demonstrates that the expression of the *LTB4R* and *LTB4R2* genes significantly negatively correlates with DNA methylation at both CpG sites cg12853742 (R = −0.4, *p* = 2.6 × 10^−6^; R = −0.21, *p* = 0.015) and cg21886367 (R = −0.45, *p* = 7.3 × 10^−8^; R = −0.24, *p* = 0.005), suggesting the upregulation of these genes in tumors with abnormal hypomethylation of their CpG island. Kaplan–Meier analysis using the TCGA-BRCA gene expression and clinical data revealed poorer overall survival for TNBC patients with an upregulated *LTB4R*. To this day, only the leukotriene inhibitor LY255283 has been tested on an MCF-7/DOX cell line, which is a luminal A breast cancer molecular subtype. Other studies compare the effects of Montelukast and Zafirlukast (inhibitors of the cysteinyl leukotriene receptor, which is different from LTB4R/LTB4R2) on the MDA-MB-231 (TNBC) cell line, with high methylation and low expression levels of LTB4R. In our study, we assess the therapeutic effects of various drugs (including leukotriene receptor inhibitors) with the DepMap gene effect and drug sensitivity data for TNBC cell lines with hypomethylated and upregulated *LTB4R/LTB4R2* genes. LY255283, Minocycline, Silibinin, Piceatannol, Mitiglinide, 1-Azakenpaullone, Carbetocin, and Pim-1-inhibitor-2 can be considered as candidates for the additional treatment of TNBC patients with tumors demonstrating *LTB4R/LTB4R2* hypomethylation/upregulation. Finally, our results suggest that the epigenetic status of leukotriene B4 receptors is a novel, potential, predictive, and prognostic biomarker for TNBC. These findings might improve individualized therapy for TNBC patients by introducing new therapeutic adjuncts as anticancer agents.

## 1. Introduction

Breast cancer (BC) is the most significant problem in modern oncology due to its prevalence among women. At least five intrinsic subtypes of breast cancer (luminal A, luminal B, HER2-enriched, basal-like or triple negative, and normal-like) have been identified using global gene expression analyses [1]. Triple negative breast cancer (TNBC) is characterized by high morbidity and mortality rates. In fact, TNBC is a class of highly heterogeneous tumors with varied molecular and clinical features. TNBC lacks estrogen receptor (ER), progesterone receptor (PR), and epidermal growth factor receptor (HER2) expression. Considering the status of ER/PR/HER2 receptors, the therapeutic effects of anticancer agents are greatly reduced [2]. Consequently, to date, TNBC cases are mainly managed by chemotherapy, which remains the standard of care. Experiments with relevant cell lines demonstrate sensitivity to different drugs, assuming promising outcomes for TNBC treatment. In vivo studies on MDA-MB-453, Sum185PE, and CAL-148 cell lines show high sensitivity to the androgen receptor (AR) antagonist bicalutamide. One of the newest therapeutic approaches is antibody–drug conjugates (ADCs). They represent highly selective monoclonal antibodies conjugated to cytotoxic agents and are designed to deliver cytotoxic drugs to antigen-expressing tumor cells [3]. Several ADCs, such as IMMU-132, CDX-011, and SGN—LIV1A, have demonstrated significant results in TNBC. *TROP2* ADCs significantly improve the prognoses of patients and increase the partial complete response (pCR) rate. In 2020, the FDA granted accelerated approval to IMMU-132 (sacituzumab govitecan), a Trop-2-directed ADC, for patients with unresectable, locally advanced, and metastatic TNBC who received two or more prior lines of therapy for metastatic disease [4]. Genomic-based targeted therapies for somatic alterations in TNBC require additional research to fully understand the value of the prognostic and predictive implications of these signatures. Targeting genetically altered signaling pathways has not yielded significant improvements in outcomes in various clinical trials when inhibitors were used alone. It was suggested that a combined therapeutic strategy, such as the combination of EGFR and mTOR inhibitors, is a reasonable choice for the treatment of TNBC, and recent experiments on TNBC cells demonstrated significant downregulation of cell cycle regulators after exposure to combined treatment [5], assuming that the combination of the EGFR inhibitor gefitinib and the mTOR inhibitor everolimus may achieve an anti-tumor effect similar to that of a single drug by reducing the drug dose [6]; yet, these are still encouraging laboratory research results. Some success with molecular targeting therapy in TNBC was achieved with poly (ADP-ribose) polymerase inhibition and immune checkpoint inhibition. PARP inhibitors (PARPi) improve the overall response rate (ORR), progression-free survival (PFS), and overall survival (OS) in combination with DNA-damaging chemotherapy [7]. The TNBC subtype is associated with a higher number of tumor-infiltrating lymphocytes (TILs), which suggests a response to immunotherapy with pembrolizumab (targets *PD1*) and atezolizumab/durvalumab (targets *PDL1*) and an improvement in the OS and PFS rates. In preclinical studies, epigenetic therapies that target DNA methylation or post-translational modifications of histone proteins are being investigated in order to treat TNBC. It was demonstrated that the combination of PARPi and DNMT inhibitors increase PARPi efficacy [8]. HDAC inhibitors enhance the effects of TNBC immunotherapy by increasing *PDL1* and *HLA-DR* expression [9]. Targeting CDK4/6 with palbociclib could improve the sensitivity of TNBC cells to paclitaxel [10]. A *TTK* inhibitor in combination with paclitaxel prevents TNBC proliferation and growth [11]. Many therapeutic agents and monoclonal antibodies produced poor therapeutic outcomes or are currently under investigation [2,3,12], which makes it relevant to continue the search for novel, predictive, and prognostic biomarkers for TNBC in order to improve individualized therapy for this type of cancer.

Eicosanoids are a group of biologically active lipid mediators of inflammation, which include prostaglandins and leukotrienes. These organic compounds play an important role in various pathologies, including cancer [13]. Various types of eicosanoids are synthesized from arachidonic acids via cascades of enzymatic reactions. The arachidonate-5-lipoxygenase enzyme (ALOX5) directs the synthesis of arachidonic acids along the path of leukotrienes. ALOX5 metabolizes arachidonic acid to the intermediate metabolite LTA4, which is converted to LTB4 by the intermediate cytosolic enzyme LTA4 hydrolase. Leukotrienes are subsequently secreted via multidrug resistance associated protein efflux transporters [14]. LTB4 mediates its intracellular functions via binding to G protein-coupled receptors, such as *LTB4R* and *LTB4R2*. At present, the role of leukotrienes in cancer is being studied with an emphasis on the use of leukotriene receptor inhibitors as therapeutic agents [13]. Inhibitors of LTB4 receptors reduce proliferation in MCF-7/DOX breast cancer cells, and the effectiveness of these antagonists correlates with the expression of LTB4 receptors [15]. It was found that the overexpression of *LTB4R2* is associated with the synthesis of interleukin-8 and increases invasion in aggressive breast cancer cell lines [16]. *LTB4R2* was implemented as a prognostic marker for gastric cancer in an eight-gene-based prognostic score [17]. One of the leukotriene-targeting drugs, etalocib (LY293111), has reached Phase 2 clinical trials in various cancer types, such as non-small cell lung cancer, pancreatic cancer, and other solid tumors [18]. Evidence shows that the treatment of BC cell lines with the *LTB4R2* antagonist LY255283 causes dramatic apoptotic cell death [19]. We have hypothesized that the expression and/or DNA methylation of *LTB4R/LTB4R2* genes might be promising prognostic and predictive markers useful in developing individualized therapy with leukotriene receptor inhibitors for malignant tumors, including BC.

## 2. Results

### 2.1. LTB4R/LTB4R2 Genes Are Abnormally Hypomethylated in a Subset of TNBC Samples

**Figure 1:**
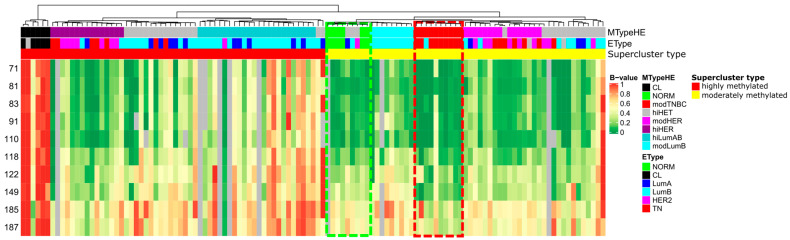
*LTB4R*/*LTB4R2* CpG island methylation heatmap. Rows represent individual CpG dinucleotides with the distance from *LTB4R* gene transcription start site (TSS) indicated on the left of each row, whereas columns correspond to samples. Red, yellow, and green colors stand for high, moderate, and low methylation levels, respectively. The moderately methylated supercluster breaks up into several smaller clusters that incorporate moderate luminal B (modLumB), moderate TNBC (modTNBC), moderate HER2-enriched (modHER), and normal tissue (NORM) samples (MTypeHE annotation bar, as assigned previously [11]). EType annotation bar provides information about immunohistochemical BC molecular subtypes.

In our recent study [20], we obtained genome wide methylation profiling results on 116 BC samples in total via reduced representation bisulfite sequencing with the XmaI restriction enzyme (XmaI-RRBS), and, using unsupervised cluster analysis, we distinguished two BC epigenetic superclusters over the CpG islands: highly and moderately methylated (Figure 1). The moderately methylated supercluster is represented by carcinoma samples belonging mainly to the three expression subtypes of BC: luminal B, HER2+, and TNBC. Among the features discriminating a moderately methylated triple negative breast cancer subtype (modTNBC) from all other BC methylotypes is the hypomethylation of a CpG island belonging to the leukotriene receptor 1 gene *LTB4R* and the leukotriene receptor 2 gene *LTB4R2* (Figure 1).

**Figure 2:**
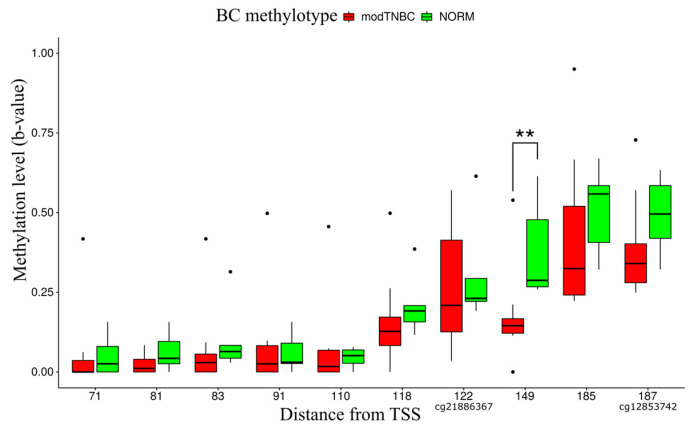
Methylation levels of CpG dinucleotides in *LTB4R*/*LTB4R2* CpG island (from the results of XmaI-RRBS genome-wide screening). The distance from *LTB4R* TSS is plotted on the x-axis, and b-value methylation level is plotted on the y-axis. Infinium Human Methylation 450 K BeadChip (HM450K) Probe IDs are denoted under the x-axis. Asterisks (**) denote positions with statistically significant b-value methylation differences according to the Mann–Whitney test.

A comparison of modTNBC samples (moderately methylated modTNBC cluster; red dashed rectangle in Figure 1) and six samples of normal breast tissue (NORM, green dashed rectangle in Figure 1) by CpG pair methylation level at different distances from the *LTB4R* gene transcription start site using the Mann–Whitney test showed statistically significant abnormal hypomethylation in breast cancer TN tissues (*p* < 0.05) at position +149 from the *LTB4R* TSS (Figure 2).

**Figure 3:**
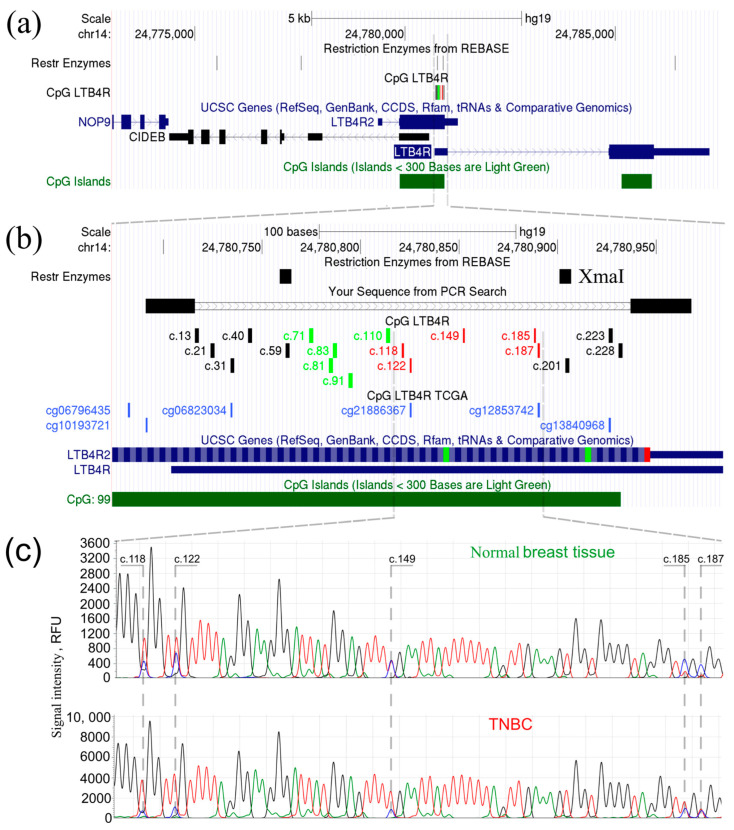
The genomic region under study and the bisulfite Sanger sequencing results. (**a**) Region of *LTB4R/LTB4R2* genes and their neighbors. (**b**) XmaI restriction endonuclease sites flank the DNA fragment where XmaI-RRBS method was used to determine methylation status of CpG dinucleotides. Cytosines within CpG pairs with indicated distance from the TSS (track “CpG LTBR4”) are colored red for hypomethylated in TNBC vs. normal breast tissues, green for the unaltered status in TNBC, and black for those not assessed by XmaI-RRBS. “Your Sequence from PCR Search” track demonstrates the position of a PCR product, with the flanking primers and an insert, used for bisulfite Sanger sequencing. In the “CpG LTBR4 TCGA” track, the CpG dinucleotides available for analysis using HM450K microarray are shown in blue. (**c**) Intensity of cytosine signal relative to thymine in TNBC is lower than that in normal breast tissue for all CpG dinucleotides studied by bisulfite Sanger sequencing within the area of interest. Electropherograms lines are colored red for thymine, blue for cytosine, black for guanine and green for adenine.

To confirm the abnormal hypomethylation status in the CpG island of *LTB4R*/*LTB4R2* genes in TN breast cancer tissues, bisulfite Sanger DNA sequencing was performed on tumor and normal tissues. Figure 3 depicts the genomic area under study: (a) a zoomed-in area with the indicated positions of the XmaI restriction endonuclease recognition sites, (b) CpG dinucleotides available for examination using XmaI-RRBS and an HM450K microarray, and (c) electropherograms of the bisulfite Sanger DNA sequencing of normal and tumor tissues.

Taking into account that the accuracy of the quantitative estimations using Sanger sequencing does not exceed 25%, the levels of cytosine methylation in CpG dinucleotides were assessed visually based on the results of bisulfite sequencing: a decrease in the intensity of cytosine peaks relative to thymine at the same position was considered as confirmation of abnormal hypomethylation. A decrease in the cytosine peak signal intensity in the tumor tissue of the studied TNBC group compared to normal breast tissue was evident, which is strongly associated with ectopic gene expression.

### 2.2. High LTB4R Expression Is Associated with Poor Prognosis in TNBC

**Figure 4:**
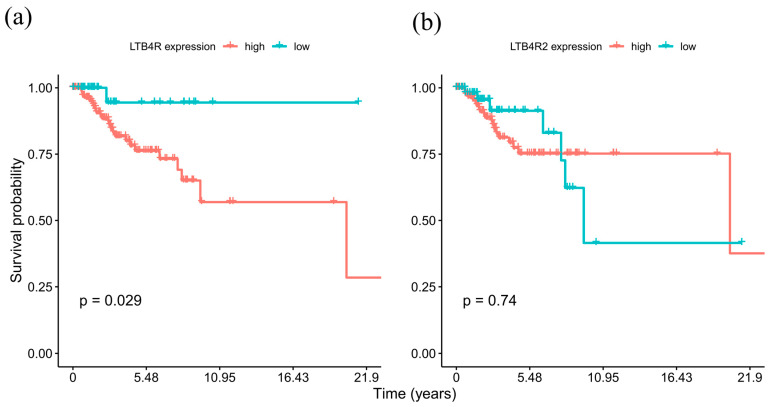
Kaplan–Meier estimator for TNBC patients with low and high (**a**) *LTB4R* gene expression and (**b**) *LTB4R2* gene expression. Log-rank test revealed significantly lower survival probability in high *LTB4R* gene expression group but no statistical difference in *LTB4R2* groups.

We analyzed the TCGA BC expression dataset by dichotomizing the RNA-seq gene expression data using maximally selected rank statistics for determining the optimal threshold in order to assess the association of *LTB4R* and *LTB4R2* expression levels with breast cancer patient survival. Figure 4 shows Kaplan–Meier survival curves for cases with high and low expression of *LTB4R* and *LTB4R2* genes.

The high-*LTB4R* group had a significantly (*p* = 0.029) shorter median overall survival (OS) of 20.42 years as compared to the group with low *LTB4R* expression, with still more than 50% survivors at the last time point. Conversely, survival analysis for the high-*LTB4R2* and low-*LTB4R2* expression groups (Figure 4b) revealed no statistically significant difference in OS (*p* = 0.74). These results suggest that *LTB4R* gene expression can serve as a potential prognostic marker in TNBC cases.

### 2.3. LTB4R and LTB4R2 CpG Methylation Levels Negatively Correlate with mRNA Expression

**Figure 5:**
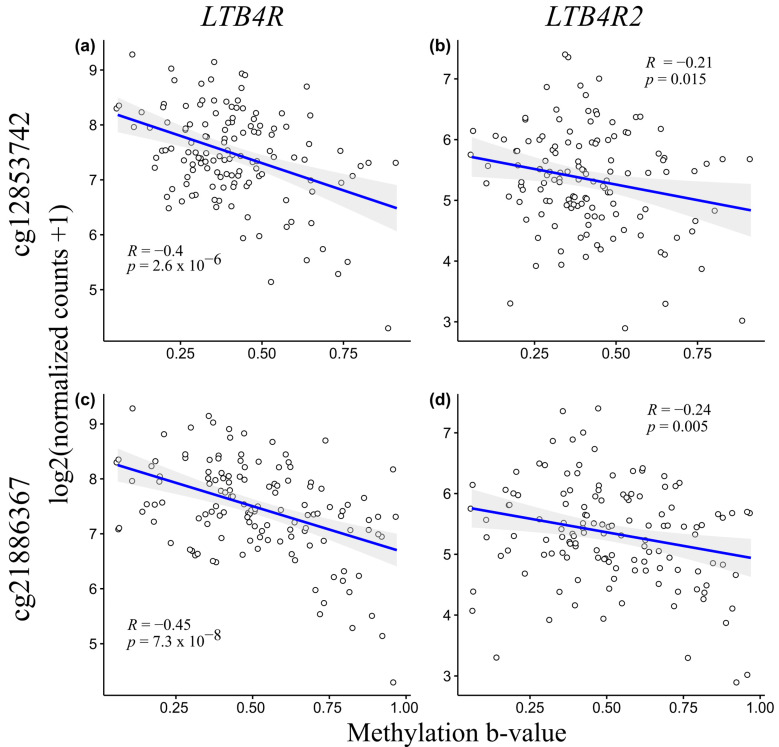
Comparison of mRNA expression and DNA methylation of *LTB4R/LTB4R2* genes assessed with two HM450K probes. X-axis denotes methylation b-value; y-axis denotes mRNA expression. Correlations between (**a**) methylation at cg12853742 and *LTB4R* expression, (**b**) methylation at cg12853742 and *LTB4R2* expression, (**c**) methylation at cg21886367 and *LTB4R* expression, and (**d**) methylation at cg21886367 and *LTB4R2* expression.

In order to assess the statistical relationship between gene expression and methylation levels of the *LTB4R*/*LTB4R2* genes, we carried out a correlation analysis using the RNA-seq expression and methylation data level 3 from the TCGA dataset with further visualization via scatterplots to assess the possible regulatory effect of DNA methylation on mRNA expression. For this purpose, HM450K methylation microarray probes with genomic coordinates that fall within the region tested using the XmaI-RRBS assay were selected. The TNBC samples (n = 130) were taken into account in relation to the benefit of having data on the methylation b-values and expression normalized counts. Figure 5 shows scatterplots of the gene expression counts and methylation b-values using probes that target cg21886367 and cg12853742.

According to the Chaddock scale, the probes that targeted cg12853752 and cg21886367 exhibited a moderate but significant negative correlation (R = −0.4, *p* = 2.6 × 10^−6^ and R = −0.45, *p* = 7.3 × 10^−8^, respectively) with *LTB4R* gene mRNA expression, whereas for the *LTB4R2* gene, we observed a weak but significant negative correlation with the methylation b-values (R = −0.21, *p* = 0.015 and R = −0.24, *p* = 0.005 respectively). These results suggest that the abnormal hypomethylation of the *LTB4R* and *LTB4R2* genes can be associated with their upregulated expression.

### 2.4. Leukotriene Receptor Inhibitors as Potential New Therapeutic Agents for TNBC

**Figure 6:**
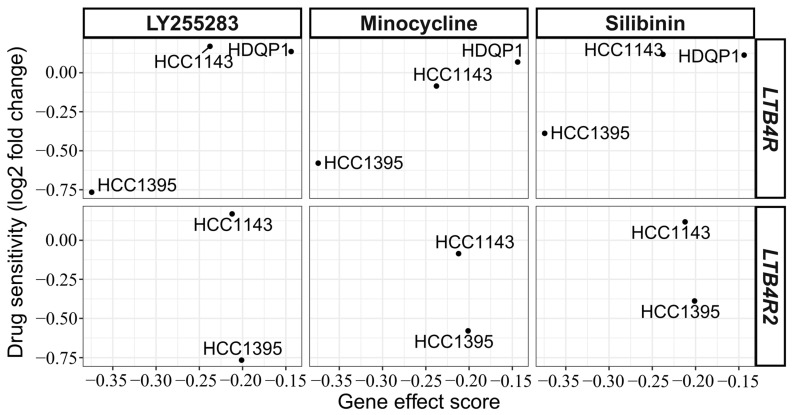
Relationship between drug sensitivity (y-axis) and *LTB4R/LTB4R2* gene effect score (x-axis). Gene effect >0 corresponds to increased cell proliferation/survival, and values < 0 correspond to decreased cell proliferation/survival. Log2 fold change drug sensitivity values > 0 indicate that the treated cells grew more readily than control cells, and values < 0 correspond to the cells that were more sensitive to treatment.

To check for a possible association between various leukotriene receptor inhibitors and the depletion of *LTB4R/LTB4R2* in TNBC cell lines with high *LTB4R/LTB4R2* mRNA expression and low DNA methylation levels, we compared primary screen values of drug sensitivity from the PRISM Repurposing dataset and cell dependency values from DepMap RNAi screen data (Figure 6). Based on the results, LY255283 exerted the best desirable effect on the HCC1395 cell line, whereas Minocycline and Silibinin exhibited slightly less efficiency in same cell line (Table S1 contains drug sensitivity/gene effect score pairs for all leukotriene receptor inhibitors).

In order to select new potential therapeutic agents, we used drug sensitivity values from the PRISM research and gene effect scores, which we mentioned above. Using Pearson correlation, we revealed potential adjuncts to the main therapy for TNBC patients. (Table 1; Table S2 contains the Pearson correlation coefficient along with the standard error (SE) and mechanisms of action for all of the possible drug sensitivity and gene effect score combinations).

## 3. Discussion

Cancer is considered a multifaceted disease that is among the leading causes of mortality. According to GLOBOCAN, in 2020, there were about 2.3 million new cases and 684.996 deaths from BC, which is the first most frequent cancer worldwide [21] and is a highly heterogeneous disease with diverse molecular and clinical characteristics. Discovering novel therapeutic targets for breast cancer is still one of the biggest challenges in omics research. Emerging technological progress in genome-wide sequencing and bioinformatics has allowed researchers to use data portals to create new therapeutic perspectives for various cancers [22]. We used the TCGA data portal, the DepMap project, and our own previously deposited genome-wide breast cancer DNA methylation screening data obtained by XmaI-RRBS from the Gene Expression Omnibus (GEO) to shed light on the possible roles of the *LTB4R/LTB4R2* genes as prognostic markers and actionable targets in TNBC [20].

One of the hallmarks of cancer is an aberration of DNA methylation patterns [23]. This common molecular alteration of the cancer genome can be used for diagnostic, prognostic, and predictive purposes [24]. An example of the predictive value of the DNA methylation of the *MGMT* gene promoter in response to temozolomide therapy in glioblastoma shows that the comprehensive characterization of DNA methylation patterns is important in both normal and cancer genomes [25]. In renal cell carcinoma, the aberrant DNA demethylation of the cancer-retina antigen recoverin is significantly linked with patient survival, making it a good prognostic biomarker [26].

Arrestin-1 is a prognostic marker of abnormal DNA demethylation. In renal cell carcinoma, survival analysis reveals that the ectopic expression of arrestin-1 is associated with the abnormal hypomethylation of its promoter in tumors and is also associated with a 70% decrease in five-year survival [27]. In breast cancer, the epigenetic regulation of the *HPGD* gene modulates the activity of the ER pathway [28].

An important instrument that can be used to discover new prognostic and predictive biomarkers is survival analysis using cancer datasets. For example, an analysis of the Cancer Genome Atlas (TCGA) Database revealed an inverse relationship between interleukin-13 receptor A1 and A2 gene expression and poor prognosis and drug resistance in subjects with glioblastoma multiforme [29]. An analysis of the BC GEO dataset revealed 21 prognostic genes that were affected by DNA methylation in the ER/HER2 subtype [30]. Notably, some of these are related to immune functions, which are in our area of interest; e.g., regarding paracrine signaling in cancer cells, LTB4 is linked with inducing and activating immune cells in the tumor microenvironment [14,31].

In this paper, we report abnormal hypomethylation of a promoter region adjacent to the *LTB4R/LTB4R2* genes in a subset of TNBC samples that is associated with the upregulation of these genes and identify abnormal DNA hypomethylation at this region as a prognostic and predictive marker with respect to leukotriene receptor inhibitors as anticancer drugs.

We demonstrate that the mRNA expression of the *LTB4R/LTB4R2* genes significantly and negatively correlates with DNA methylation at HM450K probes that target cg12853752 (*LTB4R*: R = −0.4, *p* = 2.6 × 10^−6^; *LTB4R2*: R = −0.21, *p* = 0.015) and cg21886367 (*LTB4R*: R = −0.45, *p* = 7.3 × 10^−8^; *LTB4R2*: R = −0.24, *p* = 0.005), which can be a key to upregulation of expression in cases with abnormal hypomethylation. It is noteworthy that the *LTB4R* gene exhibits only moderate but significant correlation, and the reason for this may be the location of the assessed CpG pairs relative to the gene’s transcription factors.

As expected, upregulated *LTB4R* is associated with poor prognoses in patients with TNBC. Previously, for another tumor type, e.g., clear cell renal cell carcinoma, Zhang et al. [32] presented high *LTB4R* expression as an important cancer marker and possibly a highly specific target associated with a poor prognosis due to the promotion of cancer cell proliferation, migration, development, and progression via the Akt/mTOR pathway. Pan-cancer bioinformatic analysis revealed that higher *LTB4R* expression was associated with poor relapse-free survival (RFS), poor OS, and poor distant metastasis-free survival (DMFS) in breast cancer datasets without segregation into molecular subtypes [33]. We performed a survival analysis of TCGA-BRCA RNA-seq expression data in TNBC samples coupled with clinical information, and the results show poor OS in a group with higher *LTB4R* gene expression (*p* = 0.029). One of the reasons for poor OS is that ectopic expression of *LTB4R/LTB4R2* generates reactive oxygen species (ROS) [34]. ROS are well-recognized for their ability to increase cell proliferation, DNA damage, and survival, leading to tumor growth in aggressive cancers, such as TNBC [35]. ROS production is dependent on nicotinamide adenine dinucleotide phosphate oxidase (NOX) complexes [34]. In TNBC, NF-kB signaling cascades, which are related to invasion, are regulated by the LTB4R2-NOX-1 pathway, leading to the production of IL-6 and IL-8 [16,36,37]. This evidence suggests that using leukotriene inhibitors individually or in combination with NOX inhibitors is a viable therapy to improve OS for TNBC patients and also suggests that the methylation/expression of the leukotriene receptor gene *LTB4R* is a possible prognostic marker in TNBC.

According to the DepMap data portal, the HCC1395 TNBC cell line is sensitive to LY255283 (R = 0.90), Minocycline (R = 0.98), and Silibinin (R = 0.91). While LY255283 is a leukotriene B4 receptor antagonist, Minocycline and Silibinin both target ALOX5, which plays a key role in the synthesis of leukotrienes from arachidonic acid [38]. Minocycline hydrochloride improves the blood–brain barrier, prevents the extravasation of breast cancer cells, and can be coadministered with doxorubicin-cyclophosphamide therapy without a loss of efficacy [39,40]. Silibinin is a flavonoid antioxidant from milk thistle with the potential to inhibit growth, arrest the cell cycle, and induce of apoptosis in several types of cancer cells, including BC [41]. Treatment with Silibinin sensitizes chemoresistant cells to chemotherapeutic agents [42]. Any of the above medications can possibly be used as an additional treatment to reduce the expression of the *LTB4R* gene and improve the OS rate in TNBC patients with hypomethylation/overexpression of *LTB4R*. The use of such antagonists in the treatment of patients whose molecular profile is similar to the HCC1395 cell line can be envisioned.

The Pearson correlation with drug sensitivity values and gene effect scores revealed the highest positive correlation with the top five compounds: Piceatannol (R = 0.99), Mitiglinide (R = 0.99), 1-Azakenpaullone (R = 0.99), Carbetocin (R = 0.99), and Pim-1-inhibitor-2 (R = 0.99). Piceatannol is a stilbenoid found in red wine, grapes, and sugar cane; it was shown to induce PD-L1 expression in the Cal51 (TNBC) and SW620 cell lines [43]. Piceatannol administration can improve OS and PFS in TNBC patients because its effect is likely similar to that of atezolizumab/durvalumab but with fewer adverse effects [44]. Additionally, 1-Azakenpaullone is an inhibitor of glycogen synthase kinase-3 beta (GSK-3β), which is a tempting target for cancer treatment [45]. Inhibition of GSK-3β leads to a decreased survival of breast cancer cells and attenuates their sensitivity to chemotherapy [46]. The overexpression of GSK-3β is associated with a poor OS prognosis in TNBC [47]. GSK-3β is a multifunctional serine and threonine kinase and a downstream effector of the PI3K/Akt pathway [48]. Combining GSK-3β inhibitors with Akt pathway inhibitors may result in improving OS and PFS for TNBC patients. Carbetocin acts as an agonist to the oxytocin receptor OTR. OTR reduction promotes cancer progression, whereas increased levels of OTR may decrease breast cancer proliferation. To this day, there are no data to support the prognostic/predictive value of OTR [49]. The *PIM-1* gene is upregulated in TNBC and is associated with a poor prognosis and reduced RFS and PFS in patients with the TNBC subtype. PIM1-inhibitor-2 may result in therapeutic benefits when used with standard-of-care drugs [50]. A study has not been conducted regarding the use of Mitiglinide (the Meglitinide class of blood-glucose-lowering drugs for type 2 diabetes); however, type 2 diabetes-related drugs, such as metformin, have been included in 37 trials (Phases I, II, and III) and have been associated with increased recurrence and survival rates in patients with TNBC [51,52]. Theoretically, there is a possibility of drug repurposing with any of the aforementioned substances, since they all have the potential to improve various survival rates, and their action has been demonstrated in TNBC cells with *LTB4R* overexpression. It is important to pay close attention to our selected ALOX5 and leukotriene inhibitors as well as the newly discovered therapeutic agents Piceatannol, 1-Azakenpaullone, and PIM-1-inhibitor-2. These agents might be combined with the most common doxorubicin-cyclophosphamide regimen for TNBC patients to increase sensitivity to chemotherapy. Altogether, database analysis suggests that the aforementioned drugs might be considered as potential adjunctive therapies to improve the prognosis of TNBC patients with upregulated expression and abnormal hypomethylation of the *LTB4R/LTB4R2* genes.

Researchers and clinicians will be able to use our results to develop simple and cost-effective laboratory PCR or qPCR tests to determine the methylation status of the promoter region of the *LTB4R* gene and to indirectly identify patients with increased expression of this gene in triple-negative breast tumors. This would be an easy way to select such patients for clinical trials aimed at improving individualized TNBC therapy using introducing leukotriene receptor inhibitors and newly discovered therapeutic agents as drug repositioning for TNBC patients. To date, no useful assays are available for the assessment of leukotriene B4 receptor methylation status, and most of the studies use either the MDA-MB-231 cell line (TNBC with high *LTB4R* gene methylation) [53] or pan-cancer pure bioinformatic analysis of *LTB4R* expression data from microarray or RNA-seq experiments [33].

It is important to note some limitations and strengths of this study. The most significant limitation of our study is the lack of an assessment of LTB4R/LTB4R2 gene/protein expression status either by qPCR or by immunostaining. As a surrogate, we used high quality molecular data from the TCGA consortium and the DepMap portal. Futhermore, a more detailed clarification of how *LTB4R* regulates modTNBC development is required in order to obtain sufficient evidence for the application of new therapeutic agents to mainline therapy in TNBC. A larger cohort of modTNBC samples should be investigated in order to distinguish the clinicopathological characteristics of a modTNBC subtype as well.

## 4. Materials and Methods

### 4.1. Study Design

Initially, we performed a genome-wide DNA methylation assay on a collection of 110 breast carcinoma and 6 normal breast tissue samples by XmaI-RRBS in order to provide an unbiased epigenetic BC subtype classification [20]. Here, we have used a previously obtained dataset in order to assess differential DNA methylation within the *LTB4R/LTB4R2* CpG island. From the dataset, we selected 10 modTNBC subtype samples with the most pronounced hypomethylation of this region and subjected them to bisulfite Sanger sequencing, along with 6 normal breast tissue samples. We used Sanger sequencing to validate (epi)genetic variants identified by NGS (XmaI-RRBS), which is our general practice in research and diagnostics. In DNA methylation analysis, a well-known approach that combines PCR amplification of the bisulfite-modified DNA with the subcloning of the amplicons into plasmids followed by transformation into bacteria and subsequent Sanger sequencing of several clones was used to obtain detailed information about the status of each CpG site within a region. The published protocols for bisulfite sequencing of cloned alleles state that this is the only available technique aside from high-throughput bisulfite sequencing approaches that provides allele-specific methylation status at single nucleotide resolution [54]. We have already performed high-throughput bisulfite sequencing using XmaI-RRBS, which in vitro recapitulates bisulfite sequencing of cloned alleles, but we have further elaborated standard bisulfite Sanger sequencing for qualitative validation of differential methylation only. Yet, taking into account analysis artifacts that may be produced when conventional software is used to present Sanger sequences of DNA molecules with nonequivalent nucleotide compositions, in particular, DNA molecules treated with sodium bisulfite DNA, we have used our own SeqBase software that was specifically designed to carefully analyze electropherograms based on raw data and to accurately determine the baselines in the spectral channels of individual nucleotides. Moreover, as another independent reference supporting our findings of differential methylation, we used quantitative methylation data obtained from the two CpG dinucleotides in the region available for analysis via HM450K microarray after downloading it using TCGAbiolinks package.

For differentially methylated CpG positions, we acquired TCGA-BRCA methylation data and clinical and RNA-seq expression data to perform correlation analysis of methylation/expression data followed by survival analysis with clinical and expression data. Finally, information about TNBC cell lines and drugs from DepMap portal was used to associate sensitivity of drugs with effects of *LTB4R/LTB4R2* knockout.

### 4.2. XmaI-RRBS

Genome-wide methylation analysis was conducted using the XmaI-RRBS method as previously described [55] on an Ion Torrent PGM sequencer (Thermo Fisher Scientific, Waltham, MA, USA). Briefly, the DNA was treated with restriction endonuclease XmaI, sticky ends were partially blunted with methylated cytosines using the 3′-5′ Klenow fragment to prevent self-ligation of DNA fragments and ligated adapters with methylated cytosines. Library fragments with an insert of 100–200 bp underwent bisulfite conversion using the Qiagen EpiTect Bisulfite Kit (Qiagen, Hilden, Germany). The SNaPshot Multiplex Kit (Thermo Fisher Scientific, Waltham, MA, USA) was used to block DNA fragments with chain-terminating dideoxynucleotides in order to prevent nonspecific priming of the fragments’ 3′ ends in the ensuing polymerase reaction. Next, the carrier RNA utilized in the EpiTect Bisulfite Kit procedure was eliminated using RNase A (Sigma-Aldrich, St. Louis, MO, USA), and any leftover ddNTPs were dephosphorylated using alkaline phosphatase (SibEnzyme, Novosibirsk, Russia). Final libraries were amplified by PCR and sequenced. Raw data were processed with standard Ion Torrent Suite software, followed by Bismark [56] software to align obtained reads to GRCh37/hg19 genome and acquire methylation level of CpGs.

### 4.3. Data Collecting

XmaI-RRBS results are available at NCBI GEO portal (GEO ID: GSE122799). The data represent methylation values and clinical information for 110 breast cancer and 6 normal tissue samples.

RNA–seq, Illumina HumanMethylation 450K, and clinical data were downloaded using TCGAbiolinks package [57]. Our final dataset consisted of 190 samples with the TNBC molecular subtype, *LTB4R/LTB4R2* gene expression status, methylation values of *LTB4R/LTB4R2* at HM450K using probes targeting cg12853742 and cg21886367, and patients’ survival information.

To investigate the effects of drugs, including leukotriene receptor antagonists, we used data from the DepMap portal [58]. In order to make use of this approach, we selected cell lines derived from TNBC that were characterized by high levels of expression and low levels of methylation of the *LTB4R/LTB4R2* genes. A total of 7 cell lines (HCC1143, HCC1187, HCC1395, HCC1569, HCC2157, HCC70, and HDQP1) showed similar expression behaviors and methylation levels for *LTB4R/LTB4R2* genes. Gene effect scores and drug sensitivity values were used in DepMap data explorer module.

The study was conducted according to the guidelines of the Declaration of Helsinki and was approved by the Institutional Ethics Committee of the Research Centre for Medical Genetics (the approval number is 2017-6/4).

### 4.4. Bisulfite Sanger Sequencing

Bisulfite Sanger sequencing was performed for 10 TNBC and 6 normal tissue samples to confirm cancer-associated abnormal hypomethylation of *LTB4R/LTB4R2* genes.

DNA extraction was performed using the standard phenol/chloroform method after proteinase K treatment of the tissues. DNA sodium bisulfite treatment and bisulfite PCR protocols were performed as reported previously [26].

Bisulfite PCR primers complementary to the CpG dinucleotides of interest were designed using MethPrimer portal (http://www.urogene.org/methprimer/index.html, accessed on 1 November 2023); further testing for common SNPs was performed, and MFEprimer 3.0 was used (https://www.mfeprimer.com/, accessed on 1 November 2023) to check for self-dimers, cross-dimers, and hairpins.

Electropherograms obtained from bisulfite Sanger sequencing were analyzed using SeqBase software (http://www.epigenetic.ru/projects/seqbase, accessed on 1 November 2023). SeqBase software was designed to carefully analyze electropherograms based on raw data and to accurately determine the baselines in the spectral channels of individual nucleotides. This is important for the analysis of sequencing results with nonequivalent nucleotide compositions, in particular, for sequences of DNA molecules treated with sodium bisulfite DNA.

The primer sequences used in bisulfite PCR developed in the present study were 5′-ggttttggttttttttagttttag-3′ for LTB4RbisF and 5′-aacaaattataaaatctactatcaaaaatc-3′ for LTB4RbisR. The amplification conditions for bisulfite PCR in this study are described below. Bisulfite PCR was performed in 25 μL reaction volume containing 0.1–0.2 µg of genomic DNA, 2.5 µL of a PCR buffer (10 × 670 mM Tris-HCl, pH 8.8, 25 °C), 166 mM ((NH_4_)_2_SO_4_; 0.1% Tween-20), 180 µM of each dNTP, 2.0 mM MgCl_2_, 5 pM of each primer, 1U of Taq DNA polymerase, and deionized water to a final volume. An initial 5 min denaturation step at 95 °C was followed by cycling between 95 °C for 40 s, 54 °C for 40 s, and 72 °C for 40 s for a total of 33 times, with a final 10 min elongation step at 72 °C under a layer of mineral oil. The size of the PCR products was 277 bp.

Methylation-specific Sanger sequencing was carried out according to the protocol reported previously [26].

Dye-labeled products were purified using ethanol precipitation, which included 20 mg glycogen (Roche) as an inert carrier to aid recovery following the standard protocols.

All samples were resuspended in Hi-Di™ Formamide and sequenced on the Applied Biosytems 3500 DNA Analyzer using the 50 cm Applied Biosystems Capillary Array and POP-7™ Performance Optimized Polymer.

### 4.5. Statistical Analysis and Visualization

Mann–Whitney U test and Pearson correlation test were performed using standard R 3.6.3 functions. Kaplan–Meier estimator was used to compare overall survival time in different RNA-seq expression groups, with *survival* package. *LTB4R* and *LTB4R2* expression was delineated using maximally selected rank statistics as implemented in *maxstat* R package and *survminer* package. The null hypothesis versus alternative hypothesis was tested by the log-rank (Mantel–Cox) method. A *p*-value lower than 0.05 was considered as a significant statistical difference between survival curves.

## 5. Conclusions

Data from genome-wide XmaI-RRBS bisulfite sequencing, TCGA-BRCA, and DepMap projects were used to evaluate the expression levels, status of methylation, and prognostic value of the leukotriene receptor genes *LTB4R* and *LTB4R2*. A correlation analysis of the methylation and expression of *LTB4R*/*LTB4R2* revealed positions of the CpGs in the promoter region that may contribute to the overexpression of these genes. Upregulation of *LTB4R*/*LTB4R2* in a subset of TNBC samples suggests that the expression levels of these receptors may be employed in the future as a marker of tumor sensitivity to leukotriene receptor inhibitors. There are a number of candidates for drug repurposing in TNBC, including LY255283, Silibinin, Piceatannol, Mitiglinide, 1-Azakenpaullone, Carbetocin, and PIM-1-inhibitor-2. All of these drugs show potential therapeutic effects, depending on *LTB4R*/*LTB4R2* expression and the methylation landscape. The fine mapping of abnormal DNA methylation adjacent to the *LTB4R* and *LTB4R2* genes reported in this study enables the development of a grounded methylation-sensitive PCR laboratory test to indirectly identify tumors expressing ectopic leukotriene B4 receptors and that potentially respond to these inhibitors, which is more practical in research and clinical applications than assessments of gene expression. Future studies will require more samples for genome-wide bisulfite sequencing and a clarification of the TNBC subtype demonstrating abnormal hypomethylation of *LTB4R*/*LTB4R2*, a clarification of the clinical and pathological characteristics of this subtype, an assessment of LTB4R status by immunostaining, and other relevant approaches.

## Figures and Tables

**Table 1:** Top 5 potential adjuncts to TNBC main therapy, R—correlation coefficient, SE—standard error, MOA—mechanism of action.

Drug	MOA	R	SE
PICEATANNOL	SYK inhibitor	0.99	3.60 × 10^−5^
MITIGLINIDE	Insulin secretagogue	0.99	0.0002
1-AZAKENPAULLONE	Glycogen synthase kinase inhibitor	0.99	0.001
CARBETOCIN	Oxytocin receptor agonist	0.99	0.001
PIM-1-INHIBITOR-2	PIM kinase inhibitor	0.99	0.002

## Data Availability

XmaI-RRBS genome-wide DNA methylation results are available at GSE ID GSE122799. TCGA data were downloaded using the TCGAbiolinks package, and DepMap data were used as implemented in the DepMap data portal. Any code written in R is available upon request.

## Supplementary Materials

The following supporting information can be downloaded at: https://www.mdpi.com/article/10.3390/ijms242417343/s1.
